# The Advance of In Silico Evidence to Transform Pediatric Drug Development for Rare Diseases

**DOI:** 10.1002/psp4.70139

**Published:** 2025-11-18

**Authors:** Jane Knöchel, Ping Zhao, Rajat Desikan, Jiawei Zhou, João A. Abrantes, Lutz Harnisch

**Affiliations:** ^1^ InSilicoTrials Technologies Spa Trieste Italy; ^2^ Gates Foundation Seattle Washington USA; ^3^ Clinical Pharmacology Modelling & Simulation GSK Stevenage UK; ^4^ Division of Pharmacotherapy and Experimental Therapeutics, School of Pharmacy University of North Carolina at Chapel Hill Chapel Hill North Carolina USA; ^5^ Roche Pharma Research and Early Development, Pharmaceutical Sciences Roche Innovation Center Basel Basel Switzerland; ^6^ Research & Preclinical Development (R&PD) Regeneron Pharmaceuticals, Inc. Tarrytown New York USA

Rare diseases (RDs)—defined in the U.S. as those affecting fewer than 200,000 people and in the EU as fewer than 1 in 2000—represent a persistent unmet need. These differing definitions contribute to variation in reported numbers: the U.S. recognized over 7000 rare diseases impacting 25–30 million people (https://www.fda.gov/patients/rare‐diseases‐fda) while the EU estimates around 36 million affected individuals (https://www.ema.europa.eu/en/human‐regulatory‐overview/orphan‐designation‐overview). Most manifest early in life and progress relentlessly (around 70% [https://www.rarediseasesinternational.org/living‐with‐a‐rare‐disease/]), yet fewer than 5% currently have approved therapy [1]. Pediatric rare diseases amplify every obstacle of drug development: small and heterogeneous populations, ethical constraints and limited usefulness of conventional clinical trials.

Recognizing this urgency, initiatives such as the FDA's Rare Disease Innovation Hub and the LEADER 3D Program (https://www.fda.gov/about‐fda/accelerating‐rare‐disease‐cures‐arc‐program/learning‐and‐education‐advance‐and‐empower‐rare‐disease‐drug‐developers‐leader‐3d) aim to accelerate the development of medicines. Still, as highlighted in Michelle Werner's ASCPT 2025 State‐of‐the‐Art Lecture (https://ascpt2025.eventscribe.net/agenda.asp?BCFO=&pfp=BrowsebyDay&fa=&fb=&fc=&fd=&all=1), attention alone is not enough—innovation requires translation into action. Today, the growing availability of large‐scale biological datasets and advanced modeling offers that opportunity. Pharmacometrics and systems pharmacology can transform sparse data into quantitative insights, enabling virtual exploration of therapies and supporting confident decision‐making even in the absence of large trials.

A recent review by Chen et al. outlines the distinct challenges of pediatric RDs [2]—slow disease progression, limited natural‐history data, genetic and phenotypic heterogeneity, and uncertain surrogate endpoints. These challenges call for a change in the mindset of conventional drug development, which is based on evidence generation through an extensive clinical program including multiple clinical trials.

Designing clinical trials for RDs, particularly those with genetic origins, presents unique challenges due to the difficulty in demonstrating immediate clinical improvement. Since resolving the root cause is often unattainable, the primary goal of most current RD treatment is typically to prevent disease progression rather than to elicit a rapid clinical response. This necessitates a deep understanding of the disease's progression timeline and the ability to model outcome metrics over time. Proof‐of‐concept (PoC) trials for RD often focus on detecting any treatment response—typically a binary outcome—using high‐dose strategies to maximize the chance of observing an effect. However, predicting responses across a range of doses requires intricate knowledge of the underlying biological mechanisms, including how specific pathways are influenced and how these changes translate into measurable clinical outcomes.

Model‐informed drug development (MIDD) approaches—spanning pharmacometrics, quantitative system pharmacology (QSP), and machine learning (ML)—offer a transformative means to address the intrinsic challenges for pediatric RD. These computational tools are routinely employed in modern‐day drug development to enhance our understanding of the disease and a drug's pharmacology, and to support decisions throughout the continuum of the drug development life‐cycle, including the development and approval of drugs for pediatric RDs [3].

This themed issue in CPT: Pharmacometric and Systems Pharmacology offers perspectives on the role and impact of MIDD approaches that are advancing innovative treatment of pediatric patients with rare diseases.

## 1 Advances in Computational Tools and Virtual Trials

Computational tools are now central to modern R&D research. They allow virtual design of trials, mechanistic exploration of therapies, and automated synthesis of fragmented knowledge—compressing development timelines and improving decision quality.

Duchenne Muscular Dystrophy (DMD), a progressive X‐linked neuromuscular disorder affecting primarily boys [4], exemplifies the challenges of limited patient populations, phenotypic heterogeneity, and ethical constraints in pediatric RD drug development. Two complementary computational tools leverage disease progression models to simulate trial scenarios before execution. The first tool is a trial simulator that helps researchers optimize trial designs—sample size, duration, and inclusion criteria—across five common DMD functional endpoints [4]. Case studies utilizing the tools demonstrated improved trial efficiency without compromising statistical power, crucial amid patient recruitment challenges.

Another simulation interface incorporated machine learning‐generated virtual populations and multivariate models linking functional outcomes to imaging biomarkers [5]. Validation against recent trial data confirmed its predictive accuracy. The intuitive graphical interface enables clinical leads and MIDD experts to collaboratively explore ranges of scenarios relevant to their respective clinical drug development.

QSP frameworks are likewise advancing rapidly. Meno‐Tetang et al. highlight how QSP modeling enhances understanding of biological dynamics, informs expression kinetics and durability, and guides dose optimization while mitigating off‐target effects [6]. Applications in RNA therapeutics, vaccines, and gene and enzyme replacement therapies demonstrate that QSP models now support design, translation and lifecycle management. Building on this momentum, Saini et al. introduce an AI‐augmented QSP‐Copilot, applied to the blood coagulation system and Gaucher disease [7]—a recessively inherited lysosomal storage disorder caused by deficiency of the enzyme glucocerebrosidase, leading to accumulation of glucosylceramide [8]. The tool achieved high‐precision automated data extraction (99.1% and 100%) with minimal mechanism loss. This marks a pivotal shift toward scalable, usable QSP tools with greater impact—especially in pediatric RDs where deep biological insight is essential.

Together, these advances illustrate how computational methodologies are transforming trial planning and translational decision‐making across conditions once considered too rare or complex for rigorous study.

## 2 Model‐Informed Strategies in Pediatric Rare Diseases

As therapeutic innovation expands across pediatric RDs, model‐informed strategies are proving indispensable in overcoming the limitations of small, heterogeneous populations and fragmented clinical data. From enzyme replacement in Pompe Disease to steroid delivery in ataxia telangiectasia, and exposure‐matched dosing in conditions like DMD, HoFH, CAH, and RET‐driven cancers, pharmacometric and systems modeling have enabled tailored regimens, virtual cohort bridging, and mechanistic insights. These examples reflect a broader shift toward precision and efficiency in pediatric drug development—where modeling is not just a supportive tool, but a central driver of clinical decision‐making.

Pompe disease (PD) provides a striking example. PD is a rare degenerative, multisystem metabolic disorder in which a deficient α‐glucosidase enzyme leads to the build‐up of glycogen. Depending on the age of occurrence, the later one (LOPD) leads to muscle weakness and respiratory insufficiency, while the earlier‐stage disease (IOPD) and the more rare condition lead to cardiomyopathic outcomes already during the first year of life. Rachedi et al. develop a mechanistic QSP model linking biomarkers to functional endpoints and bridge virtual cohorts between late‐ and early‐onset PD patients to optimize dosing of avalglucosidase alfa [9]. This approach identified an appropriate regimen for infants with IOPD without the need for a new, larger comparative trial.

An example in patients with Ataxia telangiectasia—a neurodegenerative disease caused by biallelic pathogenic variants in the Ataxia Telangiectasia Mutated gene [10]—illustrates how population PK modeling can characterize innovative delivery systems. Ozdin et al. integrate sparse pediatric and healthy adult data to model sustained dexamethasone release via the EryDex system, predicting safe, sustained exposure for monthly infusions—supporting long‐term steroid therapy with improved adherence and reduced toxicity [10].

Rare disease trials, especially in children, often face small sample sizes, making sensitive endpoints crucial for evaluating drug efficacy. To address this, Hamdan et al. introduced an item response theory framework that jointly models clinician‐reported (SARA) and digital motor outcomes in degenerative ataxias, reducing uncertainty, boosting statistical power, and effectively decreasing sample size by around 50%–60% [11].

Across the broader landscape of pediatric rare disease drug development, exposure matching has emerged as a central modeling strategy. Pharmacokinetic/pharmacodynamic (PK/PD) modeling and extrapolation from adult data have underpinned regulatory approvals and dosing strategies for several therapies. In the case of selpercatinib, a RET‐selective tyrosine kinase inhibitor, Liu et al. employed PopPK modeling of adult and pediatric data to define a dose regimen for children as young as 2 years old, achieving adult‐equivalent exposure and supporting FDA approval [12]. In two separate studies, Bihorel et al. demonstrate that PK/PD modeling confirms evinacumab's LDL‐C–lowering efficacy across age groups in HoFH patients, with younger children predicted to benefit as much or more than older individuals at standard dosing [13], and apply population PK/PD modeling to guide dosing in children aged 6 months to 5 years, ensuring comparable or greater LDL‐C reductions relative to older cohorts [14]. The model‐informed dose was subsequently validated through pharmacodynamic outcomes in a managed access program.

For DMD, Patel et al. analyzed pooled data from six small studies spanning ages 6 months to 16 years to support a uniform weight‐based dosing regimen for eteplirsen [15]. Notably, progressive muscle mass loss—a hallmark of DMD—was not identified as a significant covariate affecting drug exposure. In another example of model‐driven personalization, Bindellini et al. developed a population exposure–response model to characterize the interplay between endogenous adrenocorticotropic hormone and cortisol dynamics in patients with congenital adrenal hyperplasia [16]. This model enables estimation of residual 21‐hydroxylase enzymatic activity and supports individualized cortisol replacement therapy across pediatric and adult populations.

Across these examples, model‐based evidence replaces what conventional trials cannot deliver, enhancing both precision and feasibility. The message is clear: in pediatric RDs, modeling is no longer an auxiliary tool—it is central to drug development and regulatory success.

## 3 Beyond Drug Development: Model‐Based Approaches in Post‐Marketing Assessment

Model‐informed approaches now well extend beyond drug development, guiding post‐marketing assessment, benefit–risk evaluation, and labelling updates. Asiimwe et al., demonstrated this potential through a model‐based meta‐analysis (MBMA) integrating data from 103 trials of trastuzumab emtansine and trastuzumab deruxtecan [17]. By combining exposure‐response modeling with a clinical‐utility index balancing efficacy and toxicity, the authors identified optimal dosing ratios that accurately predicted the approved doses—illustrating that the MIDD can inform lifecycle management and dose refinement even in data‐limited contexts.

## 4 Future Directions and Emerging Trends

The next frontier of pediatric RD research lies in merging mechanistic modeling, artificial intelligence, and regulatory innovation. Virtual cohorts, digital twins, and hybrid QSP + ML frameworks are emerging as powerful complements to traditional trials, capable of simulating biological variability and supporting individualized treatment decisions.

Sips et al. described how machine‐learning–based virtual patients can augment control arms, as in an ongoing Phase 2 study for amyotrophic lateral sclerosis where 60 ML‐generated virtual participants supplement 20 placebo subjects [18]. Mechanistic QSP‐derived virtual patients, requiring less real‐world data, have enabled pediatric extrapolation in conditions such as Gaucher disease type 1, acid sphingomyelinase deficiency, and spinal muscular atrophy. Hybrid models that combine biological constraints with data‐driven learning are increasingly gaining regulatory recognition under MIDD frameworks as viable surrogates for synthetic control arms and patient‐specific forecasting.

At a global level, Imai et al. highlighted persistent inequities in rare‐disease drug access [19]. Roughly 70% of FDA/EMA approved drugs from 2016 to 2020 remain unavailable in Japan, and many low‐income countries lack orphan‐drug policies altogether. The authors proposed combining MIDD and AI/ML approaches to accelerate cross‐regional trial bridging, improve probability of regulatory success, and shorten time to global availability. Their proposed continuum—from QSP in preclinical evaluation to PBPK and PKPD in the clinical stages and MBMA for post‐marketing—illustrates how computational tools can move the MIDD paradigm from innovation to implementation.

Collectively, these innovations point toward an increasingly integrated future: one where data, models, and policy interact seamlessly to ensure that no population is left behind.

As pointed out in the recent special issue in CPT, patients with rare and neglected diseases often endure a long, challenging, and costly path to diagnosis and appropriate treatment [1]. Collectively, the contributions in this issue underscore the accelerating convergence of mechanistic modeling, artificial intelligence, and regulatory science as the foundation for next‐generation model‐informed drug development. From virtual cohorts and hybrid digital twins to AI‐augmented QSP frameworks, these tools are reshaping how we understand biology, design trials, and extend therapeutic access—particularly in pediatric RD settings where traditional statistical and empirical methods fall short.

Building on the established practice of MIDD to support product development decisions, the advancement of tools and their novel applications under the extreme data and population challenges of PRDs is envisioned to further innovate drug development and patient access across all therapeutic areas.

## Conflicts of Interest

The authors declare no conflicts of interest.

## References

[1] A. Ramamoorthy , I. Younis , Y. F. Wen , M. Mehanna , K. M. Giacomini , and P. H. van der Graaf , “Therapeutic Orphan no More: Role for Clinical Pharmacology and Translational Science in Developing Therapeutics for Rare and Neglected Diseases,” Clinical Pharmacology and Therapeutics 116 (2024): 1363–1368. 39543918 10.1002/cpt.3474

[2] J. Chen , L. Nie , S. Lee , et al., “Challenges and Possible Strategies to Address Them in Rare Disease Drug Development: A Statistical Perspective,” Clinical Pharmacology and Therapeutics 118 (2025): 62–73. 40079686 10.1002/cpt.3631

[3] R. Krishna , A. Mitra , M. L. Zierhut , L. East , and C. Durairaj , “State‐Of‐The‐Art on Model‐Informed Drug Development Approaches for Pediatric Rare Diseases,” CPT: Pharmacometrics & Systems Pharmacology 14 (2025): 1743–1759. 10.1002/psp4.70083 PMC12625129 40697166

[4] J. Wilk , V. Aggarwal , M. Pauley , et al., “A Computational Tool to Optimize Clinical Trial Parameter Selection in Duchenne Muscular Dystrophy: A Practical Guide and Case Studies,” CPT: Pharmacometrics & Systems Pharmacology 14 (2025): 1787–1796. 10.1002/psp4.13281 PMC12625090 39600251

[5] J. Kim , J. F. Morales , S. Kang , et al., “A Model‐Informed Clinical Trial Simulation Tool With a Graphical User Interface for Duchenne Muscular Dystrophy,” CPT: Pharmacometrics & Systems Pharmacology 14 (2025): 1765–1774. 10.1002/psp4.13246 PMC12625128 39360574

[6] G. M. L. Meno‐Tetang , N. Rayad , and E. A. Chowdhury , “The Impact of QSP Modeling on the Design and Optimization of Gene Therapy Approaches,” CPT: Pharmacometrics & Systems Pharmacology 14 (2025): 1760–1764. 10.1002/psp4.70131 PMC12625082 41220317

[7] A. Saini and A. Farnoud , “QSP‐Copilot: An AI‐Augmented Platform for Accelerating Quantitative Systems Pharmacology Model Development,” CPT: Pharmacometrics & Systems Pharmacology 14 (2025): 1775–1786. 10.1002/psp4.70127 PMC12625087 41159846

[8] M. Vigan , J. Stirnemann , C. Caillaud , et al., “Modeling Changes in Biomarkers in Gaucher Disease Patients Receiving Enzyme Replacement Therapy Using a Pathophysiological Model,” Orphanet Journal of Rare Diseases 9 (2014): 95. 24980507 10.1186/1750-1172-9-95 PMC4094900

[9] F. Rachedi , R. Jreich , S. Sparks , et al., “Clinical Modeling of Motor Function to Predict Treatment Efficacy and Enable In Silico Treatment Comparisons in Infantile‐Onset Pompe Disease,” CPT: Pharmacometrics & Systems Pharmacology 14 (2025): 1797–1809. 10.1002/psp4.13287 PMC12625105 39670959

[10] D. Ozdin , L. Kheibarshekan , G. Mambrini , and P.‐O. Tremblay , “Population Pharmacokinetic Modeling and Pediatric Exposure of Dexamethasone Sodium Phosphate Encapsulated in Erythrocytes (eDSP) Administered Monthly for Treatment of Neurological Symptoms of Patients With Ataxia Telangiectasi,” CPT: Pharmacometrics & Systems Pharmacology 14 (2025): 1882–1892. 10.1002/psp4.70103 PMC12625168 40984042

[11] A. Hamdan , A. Traschütz , L. Beichert , et al., “Integrated Modeling of Digital‐Motor and Clinician‐Reported Outcomes Using Item Response Theory: Towards Powerful Trials for Rare Neurological Diseases,” CPT: Pharmacometrics & Systems Pharmacology 14 (2025): 1857–1868. 10.1002/psp4.70081 PMC12625125 40685914

[12] D. Liu and J. S. van der Walt , “Population Pharmacokinetics Modeling of Selpercatinib to Support Posology in Pediatric Patients With RET‐Altered Metastatic Thyroid Cancer or Solid Tumors,” CPT: Pharmacometrics & Systems Pharmacology 14 (2025): 1848–1856. 10.1002/psp4.70042 PMC12625185 40358020

[13] S. Bihorel , R. Dingman , J. Mendell , et al., “Population Pharmacokinetics and Exposure‐Response Modeling for Evinacumab in Children, Adolescents, and Adults With Homozygous Familial Hypercholesterolemia,” CPT: Pharmacometrics & Systems Pharmacology 14 (2025): 1823–1834. 10.1002/psp4.70016 PMC12625133 40095399

[14] S. Bihorel , R. Dingman , J. Mendell , et al., “Comparison of Model‐Predicted and Observed Evinacumab Pharmacokinetics and Efficacy in Children Aged < 5 Years With Homozygous Familial Hypercholesterolemia,” CPT: Pharmacometrics & Systems Pharmacology 14 (2025): 1835–1847. 10.1002/psp4.70017 PMC12625149 40095766

[15] Y. Patel , L. Orogun , N. Yocum , L. R. Rodino‐Klapac , and L. East , “A Population Pharmacokinetic Model to Inform Extension of the Eteplirsen Dosing Regimen Across the Broad DMD Population,” CPT: Pharmacometrics & Systems Pharmacology 14 (2025): 891–903. 40009522 10.1002/psp4.70001 PMC12072230

[16] D. Bindellini , R. Michelet , Y. Mirasbekov , et al., “Predicting Residual 21‐Hydroxylase Enzymatic Activity in Pediatric and Adult Congenital Adrenal Hyperplasia Patients: Towards Individualized Therapy,” CPT: Pharmacometrics & Systems Pharmacology 14 (2025): 1869–1881. 10.1002/psp4.70086 PMC12625145 40714914

[17] I. G. Asiimwe , N. Chtiba , S. Mouksassi , et al., “Postmarketing Assessment of Antibody–Drug Conjugates: Proof‐Of‐Concept Using Model‐Based Meta‐Analysis and a Clinical Utility Index Approach,” CPT: Pharmacometrics & Systems Pharmacology 14 (2025): 1810–1822. 10.1002/psp4.70013 PMC12625083 40040312

[18] F. Sips , M. Virgolin , G. Pasculli , et al., “Transforming Pediatric Rare Disease Drug Development: Enhancing Clinical Trials and Regulatory Evidence With Virtual Patients,” CPT: Pharmacometrics & Systems Pharmacology 14 (2025): 1739–1742. 10.1002/psp4.70096 PMC12625110 40757668

[19] Y. Imai , E. Akatsu , and S. K. Minton , “Mitigate Japan's Drug Loss With Model‐Informed Drug Development,” CPT: Pharmacometrics & Systems Pharmacology 14 (2025): 1735–1738. 10.1002/psp4.70126 PMC12625164 41076586

